# Cell Death Mechanisms in *Mycobacterium abscessus* Infection: A Double-Edged Sword

**DOI:** 10.3390/pathogens14040391

**Published:** 2025-04-16

**Authors:** Rhana Berto da Silva Prata, Roberta Olmo Pinheiro

**Affiliations:** Leprosy Laboratory, Oswaldo Cruz Institute, Oswaldo Cruz Foundation, FIOCRUZ, Rio de Janeiro 21040-360, Brazil; rhana@ioc.fiocruz.br

**Keywords:** cell death, immunopathogenesis, non-tuberculous mycobacteria

## Abstract

Infections caused by non-tuberculous mycobacteria (NTM), such as *Mycobacterium abscessus*, elicit diverse cell death mechanisms including apoptosis, necrosis, and pyroptosis, which play key roles in immunopathogenesis. NTM can manipulate these cell death pathways to evade host immune responses, ensuring their intracellular survival and persistence. Apoptosis may aid in antigen presentation and immune activation, while necrosis and pyroptosis trigger excessive inflammation, leading to tissue damage. Autophagy, a crucial cellular defense mechanism, is often induced in response to NTM infection; however, *M. abscessus* has evolved mechanisms to inhibit autophagic processes, enhancing its ability to survive within host cells. This manipulation of cell death pathways, particularly the dysregulation of autophagy and ferroptosis, contributes to chronic infection, immune evasion, and tissue damage, complicating disease management. Understanding these mechanisms offers potential therapeutic targets for improving treatment strategies against *M. abscessus* infections.

## 1. Introduction

Most non-tuberculous mycobacteria (NTM) originate from environmental sources, such as drinking water, natural water bodies, and soil. Rather than being mere contaminants [1], NTMs are natural inhabitants of these environments. They are increasingly recognized as opportunistic pathogens in humans, primarily colonizing and infecting individuals with chronic lung diseases such as cystic fibrosis, chronic obstructive pulmonary disease, and bronchiectasis, as well as those with a history of infectious lung diseases like tuberculosis [2,3,4,5,6]. Infections can also occur in genetically predisposed individuals [7,8,9,10], those with specific body morphotypes [11], individuals exposed to contaminated needles or surgical materials, and in cases of wound infections following trauma [12,13,14,15,16,17].

Current epidemiological data show a general increase in the detection of NTM throughout the world, both in the context of infections and related diseases, as well as a significant increase in the detection of infection and disease caused by the *Mycobacterium abscessus* complex in the last decade [18,19].

*M. abscessus* was isolated for the first time in 1953 [20]; since then, its nomenclature has changed several times, as has the grouping of species with high similarity, finally being called subspecies, with the presence of *M. abscessus* subspecies *massiliense*, *M. abscessus* subsp. *abscessus,* and *M. abscessus* subsp. *bolletii* [21,22,23] and grouped in the *M. chelonae–abscessus* complex [24,25,26].

During infection by *M. abscessus*, its ability to subvert host immune mechanisms was demonstrated through the induction of biofilm production and the rope effect, which inhibits bacterial phagocytosis and prolongs mycobacterial survival [27,28]. Likewise, *M. abscessus* can induce prominent levels of reactive oxygen species (ROS) and elevated levels of pro-inflammatory cytokines [29]. These mechanisms are responsible for inducing cell death during mycobacterial infection.

Cell death is a fundamental, homeostatic, and physiological process that occurs in all living beings. It has a crucial function both in the elimination of damaged or infected cells and in the regulation of the immune response. Its action ranges from embryonic development to organ maintenance and aging [30,31]. The initiation of apoptosis, autophagy, necrosis, and pyroptosis pathways is essential for immunity against many intracellular and extracellular bacteria. These cellular killing mechanisms are utilized by the infected host to restrict and eliminate bacterial pathogens [32].

Cell death processes can contribute to an effective immune response against pathogens; however, unregulated inflammation can exacerbate tissue damage caused by bacterial infections. In response, bacterial pathogens have evolved secreted virulence factors and effector proteins that manipulate cell death pathways to facilitate infection [32]. These mechanisms are tightly regulated, as uncontrolled infection-induced inflammation can be fatal [33]. In this study, we observed the dual role of cell death during *M. abscessus* infection, where it can either help control and restrict bacterial spread or contribute to dissemination and growth. This article summarizes the key types of cell death involved in *M. abscessus* infection and highlights their major consequences.

Recent studies point to the possibility of developing new therapeutic alternatives that target the host in the treatment of diseases caused by mycobacteria [34]. Understanding the cell death mechanisms modulated by *M. abscessus* could contribute to a better understanding of the target pathways and processes for proposing new chemotherapy regimens.

## 2. Cell Death in the Immune Response

### 2.1. Apoptosis: Mechanisms, Immune Regulation, and Disease Implications

Apoptosis, or programmed cell death, is a tightly regulated, energy-dependent process essential for maintaining tissue homeostasis, controlling immune cell populations, and supporting organismal development. Unlike necrosis, apoptosis allows the safe removal of damaged, infected, or unnecessary cells without eliciting an inflammatory response [35,36].

There are three main apoptotic pathways: the extrinsic pathway, triggered by external ligands (e.g., FasL, TNF) binding to death receptors like Fas and TNFR, leading to recruitment of adaptor proteins such as FADD and TRADD, which activate caspase-8 and downstream effectors like caspase-3 [37,38,39,40]; the intrinsic (mitochondrial) pathway, activated by intracellular stressors (e.g., DNA damage, oxidative stress), where pro-apoptotic Bax and Bak promote mitochondrial outer membrane permeabilization, releasing cytochrome c to form the apoptosome with Apaf-1 and procaspase-9, which activates caspase-9, followed by caspases-3 and -7 [39,40,41]; and the perforin/granzyme pathway, mediated by cytotoxic T lymphocytes and NK cells, which use granzyme B to activate caspases and cleave Bid, linking to the intrinsic pathway, while granzyme A promotes caspase-independent DNA damage [30,40,42,43,44].

Apoptosis plays a critical role in immune regulation by eliminating infected or self-reactive cells, thus preventing autoimmune disorders such as lupus and multiple sclerosis [45,46,47,48,49]. It is essential for contracting T and B lymphocyte populations after antigen clearance, mediated through the FASL/ALG2 pathway and reduced IL-2 signaling [50,51,52]. Cytotoxic T lymphocytes eliminate infected or transformed cells via perforin and granzymes, while dendritic cells phagocytose apoptotic cells and present their antigens to T cells, contributing to adaptive immune activation [53,54,55,56].

Failures in apoptotic regulation are associated with autoimmune diseases and tumor development, where defective apoptosis permits the survival of autoreactive or malignant cells [53,57,58,59,60]. Thus, beyond its homeostatic functions, apoptosis is central to immune balance and disease prevention.

### 2.2. Necrosis and Necroptosis: Mechanisms, Immune Response and Pathological Implications

Necrosis is a form of cell death triggered by severe damage such as trauma, infection, toxins, or ischemia, and is characterized by plasma membrane rupture, cell swelling, and the uncontrolled release of intracellular contents—events that elicit a strong inflammatory response [61,62,63]. Although traditionally viewed as a passive process, necrosis can also occur in a regulated manner via necroptosis, a programmed form of necrosis mediated by RIPK1, RIPK3, and MLKL, which together form the necrosome complex that disrupts membrane integrity and promotes cell lysis [64,65,66,67].

The intracellular components released during necrosis, known as DAMPs (damage-associated molecular patterns)—such as HMGB1, ATP, uric acid, and DNA fragments—are recognized by pattern recognition receptors (PRRs) like TLRs and NOD-like receptors, triggering the activation of macrophages, neutrophils, and dendritic cells [68,69,70]. These cells release pro-inflammatory cytokines (e.g., TNF-α, IL-1β, IL-6), recruit additional immune cells, and promote debris clearance. Dendritic cells link innate and adaptive immunity by presenting necrotic antigens to T lymphocytes [70].

While this inflammatory response is essential for pathogen clearance and tissue repair, excessive or unresolved necrosis may lead to chronic inflammation, tissue fibrosis, and contribute to the pathogenesis of autoimmune and degenerative diseases [63,69,71]. Necrosis—whether accidental or regulated—thus plays a dual role in host defense and disease progression, making it a critical process in both physiological and pathological contexts.

### 2.3. Autophagy: Mechanisms, Immune Functions, and Role in Cell Death

Autophagy is a conserved cellular process that degrades and recycles cytoplasmic components, maintaining homeostasis under stress conditions such as nutrient deprivation, oxidative stress, DNA damage, and infection [72]. Beyond its role in cell survival, autophagy contributes to immune defense by eliminating intracellular pathogens and modulating both innate and adaptive immune responses [68].

The process begins with the formation of a phagophore, which engulfs cellular material, forming an autophagosome that later fuses with lysosomes for degradation [72]. This mechanism is tightly regulated by nutrient-sensing pathways, notably mTORC1 (a negative regulator) and AMPK (a positive regulator) [73,74,75]. Key protein complexes such as ULK1, PI3K class III (including Beclin-1, VPS34), and ATG12–ATG5–ATG16L1 coordinate the formation and elongation of the autophagosome. The conversion of LC3-I to LC3-II marks autophagosome maturation [74,75,76,77,78].

Autophagy can be triggered by infections, cytokines (e.g., TNF, IFN-γ), and activation of pattern recognition receptors (PRRs), such as TLRs and NLRs [79,80,81]. In this context, autophagy not only degrades pathogens but also influences cytokine production and antigen presentation. For instance, *M. tuberculosis* activates autophagy to limit its own replication inside macrophages [82].

However, when excessively activated or dysregulated, autophagy can lead to autophagic (type II) cell death, characterized by uncontrolled self-digestion of vital organelles, including mitochondria and ribosomes, resulting in energy depletion, loss of protein synthesis, membrane instability, and ultimately cell death [83,84,85,86,87,88]. The accumulation of reactive oxygen species (ROS) exacerbates this process, further damaging cellular components, as observed in chronic infections such as those caused by *M. tuberculosis.* A characteristic already described for Mtb, which shows an ability to induce ROS and transit via the macrophage phagosomal compartments, is the type I IFN response, which increases the expression of 25-hydroxycholesterol [86,87].

There is ongoing debate as to whether autophagic death is a distinct pathway or occurs in conjunction with other forms of cell death. Unlike apoptosis, it does not involve caspase activation or DNA fragmentation, and unlike necrosis, it proceeds in a more regulated but equally fatal manner [35,36,89,90,91,92].

Moreover, recent evidence links autophagy to ferroptosis. Inducers of ferroptosis, such as erastin, can stimulate chaperone-mediated autophagy (CMA), which leads to the degradation of GPX4, a key enzyme that protects against lipid peroxidation. Inhibiting CMA stabilizes GPX4 and reduces ferroptotic death, suggesting an important crosstalk between autophagy and ferroptosis during oxidative stress responses [93,94,95].

Thus, although autophagy is fundamentally a cytoprotective process, its dysregulation can contribute to cell death through complex signaling networks, with significant implications for immune responses, infection control, and disease progression.

### 2.4. Pyroptosis: Mechanisms, Immune Functions, and Role in Cell Death

Pyroptosis is a pro-inflammatory form of programmed cell death that plays a key role in eliminating intracellular pathogens and activating the immune response. It is typically initiated by the detection of pathogen-associated molecular patterns (PAMPs) or damage-associated molecular patterns (DAMPs) by receptors such as NLRP3, NLRC4, or AIM2, which activate inflammasome complexes [88].

Once assembled, the inflammasome recruits and activates caspase-1, and in humans, caspases-4 and -5 (or caspase-11 in mice) also contribute by sensing cytosolic lipopolysaccharide (LPS) [89,90]. These caspases cleave gasdermin D, releasing its N-terminal fragment, which forms membrane pores, causing cell swelling and lysis [91,92]. This process also promotes the release of pro-inflammatory cytokines IL-1β and IL-18, intensifying the immune response [91].

By lysing infected cells and releasing DAMPs like ATP and HMGB1, pyroptosis prevents pathogen replication and recruits immune cells, aiding infection control [89,90,91,92,93]. While controlled pyroptosis is protective, excessive activation can lead to damaging inflammation and is implicated in inflammatory diseases [93].

Understanding pyroptosis is essential for developing therapies targeting infectious and autoimmune diseases, with the potential to reduce pathological inflammation or prevent conditions such as sepsis.

### 2.5. Ferroptosis: Mechanisms and Role in Cell Death

Ferroptosis is a regulated, caspase-independent form of cell death characterized by iron accumulation, lipid peroxidation, and failure of antioxidant defenses, leading to irreversible damage to cellular membranes [94]. A central regulator of ferroptosis is the Xc^−^ antiporter system, composed of SLC7A11 and SLC3A2, which imports cystine—a precursor for glutathione (GSH) synthesis. GSH is essential for the activity of GPX4, an enzyme that reduces lipid hydroperoxides. When GSH is depleted or GPX4 is inhibited, lipid peroxides accumulate and trigger ferroptosis [95,96]. 

Iron metabolism also plays a pivotal role. Upregulation of transferrin, TfR1, and ferritin increases intracellular iron levels. Excess free iron promotes reactive oxygen species (ROS) generation via the Fenton reaction, enhancing lipid peroxidation [95,97,98,99]. Enzymes like ACSL4 and LPCAT3 incorporate polyunsaturated fatty acids (PUFAs) into membrane phospholipids, making them more susceptible to oxidation. These substrates are further oxidized by lipoxygenases (LOXs), contributing directly to ferroptotic death [100,101,102,103]. Together, the balance of cysteine import, GSH availability, GPX4 function, and iron homeostasis defines a cell’s sensitivity to ferroptosis. Disruption of any of these pathways promotes oxidative damage to lipids and ferroptotic cell death.

## 3. Cell Death in *M. abscessus* Infection

### 3.1. Modulation of Apoptotic Pathways by M. abscessus

The interaction of *M. abscessus* with apoptotic mechanisms is complex and plays a key role in the pathogenesis and immune response during infection. This fast-growing mycobacterium can modulate apoptosis pathways to favor its survival and dissemination within the host [104]. After infection, *M. abscessus* is primarily internalized by macrophages. Pattern recognition receptors (PRRs), such as Toll-like receptor 2 (TLR2), identify components of the bacterial cell wall, triggering innate immune responses. This interaction activates signaling cascades that lead to the release of pro-inflammatory cytokines, such as TNF and IL-1β, which can influence the apoptotic pathway [105,106,107].

**Figure 1:**
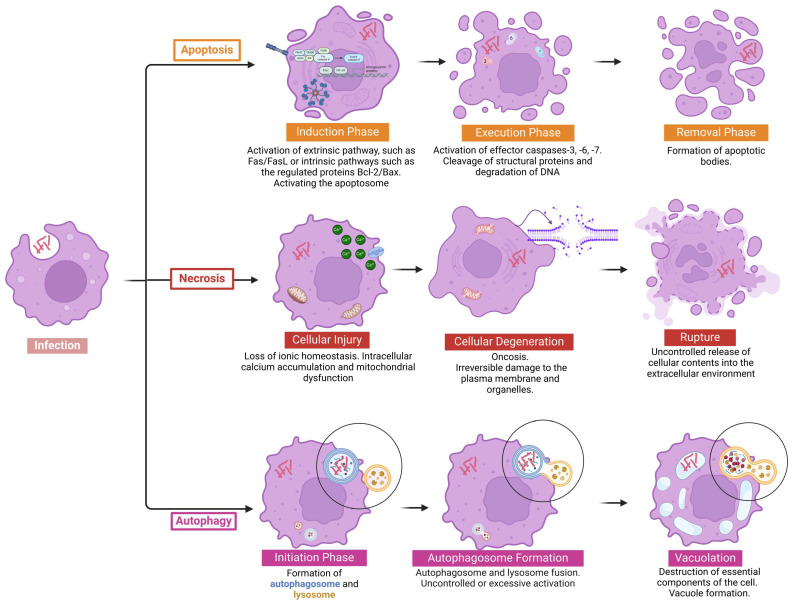
Mechanisms of cell death during *M. abscessus* infection. Following infection by *M. abscessus*, how previously described, different cell death pathways are triggered in response to the infection, highlighting key events that characterize apoptosis, necrosis, and autophagy. In apoptosis, the process begins with an induction phase, where external or internal signals (such as the activation of death receptors and the release of mitochondrial factors) recruit initiator caspases. This is followed by the execution phase, in which effector caspases cleave structural proteins and fragment DNA, ultimately leading to the formation of apoptotic bodies. Finally, during the removal phase, these fragments are systematically cleared by phagocytosis from macrophages or neighboring cells, without releasing inflammatory contents. In contrast, necrosis is marked by progressive cellular damage (cellular injury) that leads to a loss of ionic homeostasis, swelling (degeneration), and organelle dysfunction, culminating in plasma membrane rupture and the uncontrolled release of intracellular components, which triggers a strong inflammatory response. Autophagy begins during the initiation phase, when cellular stress signals, such as mTOR inhibition or AMPK activation, promote the formation of the phagophore and the recruitment of ATG proteins. In the next phase, autophagosome formation, the phagophore engulfs damaged organelles and proteins, generating the autophagosome. Upon fusion with lysosomes, the autolysosome is formed. When this process occurs at physiological levels, it facilitates the recycling of cellular components and supports cell survival. However, when it is exaggerated (vacuolation), excessive degradation of essential structures occurs, leading to cell collapse and eventual cell death by autophagy. Created in BioRender. Pinheiro, R.O. (2025) https://BioRender.com/0zr3igz.

Furthermore, infection by *M. abscessus* can modulate apoptotic pathways, including both the intrinsic (mitochondrial) and extrinsic (death receptor) pathways. *M. abscessus* can affect the mitochondrial integrity of macrophages (Figure 1). Through the generation of oxidative stress, this dysfunction results in the loss of mitochondrial membrane potential, leading to the release of cytochrome c into the cytoplasm. The release of cytochrome c allows the formation of the apoptosome, which recruits and activates caspase-9. The subsequent cascade leads to the activation of effector caspases, such as caspase-3, promoting the cleavage of essential proteins and cell death [108,109,110,111].

Additionally, cytokines released in response to infection, such as TNF, can bind to death receptors on the cell surface, such as the Fas receptor. This binding triggers the formation of a signaling complex involving adapters like FADD (Fas-associated death domain). The formation of the death complex activates caspase-8, which can initiate the cascade of executioner caspases, promoting apoptosis. In some contexts, the extrinsic pathway can also amplify the intrinsic pathway through the cleavage of Bcl-2 family proteins, inducing apoptosis [110,111,112].

*M. abscessus* has two main morphotypes: a smooth morphotype, generally associated with less inflammation, which tends to inhibit or delay the apoptosis of macrophages, allowing the intracellular survival of the bacteria and the formation of biofilms, contributing to the persistence of the infection; and a rough morphotype, which is more virulent and can induce a more intense apoptotic response, leading to the death of macrophages [29,104,113].

In this context, apoptosis can have a dual role. In a controlled manner, apoptosis can help contain the infection by eliminating infected cells without triggering exacerbated inflammation. In contrast, unregulated apoptosis can result in the failure to remove apoptotic bodies, causing the release of bacteria, facilitating their dissemination, and intensifying the inflammatory response [110,111,112,113,114]. Apoptosis of infected macrophages may function as a host defense strategy, removing compromised cells and limiting the pathogen’s replication niche. On the other hand, by inhibiting or unregulated inducing apoptotic signals, *M. abscessus* creates an environment that favors its survival and eventual dissemination [104].

During *M. abscessus* infection, apoptosis can facilitate bacterial containment by promoting phagocytosis of infected cells by neighboring macrophages, preventing the spread of the pathogen [115]. However, *M. abscessus* has developed mechanisms to inhibit apoptosis, such as modulation of the extrinsic pathway through downregulation of death receptors (Fas, TRAIL) and increased expression of antiapoptotic proteins, such as Bcl-2. This strategy allows intracellular survival of the bacteria and favors their persistence in the host [116].

While the controlled activation of apoptosis may represent an effective host defense, the ability of *M. abscessus* to alter these pathways—whether by inhibiting apoptosis to remain in a safe intracellular environment or by inducing cell death that favors its dissemination—demonstrates the complexity of this interaction and the importance of understanding these mechanisms for the development of more effective therapies.

### 3.2. Mechanisms of Necrosis Induced by M. abscessus

The induction of necrosis by *M. abscessus* is a multifactorial phenomenon that results from a combination of direct damage caused by bacterial virulence factors and an exacerbated inflammatory response from the host.

Several intrinsic factors can induce this type of cell death, such as morphotype and cord formation. The rough morphotype is particularly associated with virulence. This morphotype forms aggregated structures known as cords. The formation of these cords makes complete internalization by macrophages difficult and can lead to an overload on the host cell, resulting in cellular stress and, consequently, rupture of the plasma membrane [28,114].

Furthermore, virulence factors and cell wall components can induce necrosis in infected cells. Cell wall components, such as lipoproteins and lipooligosaccharides, can be recognized by host receptors and trigger an intense inflammatory response. This response can include the release of reactive oxygen species (ROS) and proteolytic enzymes that, in high concentrations, cause irreversible damage to cell membranes [28,115].

As stated, during infection, macrophages lead to the massive release of pro-inflammatory cytokines, such as TNF and IL-1β. Elevated levels of pro-inflammatory cytokines can lead to exaggerated activation of cell death pathways. The production of ROS and the release of degradative enzymes, such as metalloproteinases, contribute to the destabilization of the plasma membrane, culminating in necrosis. In this context, the cell cannot maintain its integrity, and rupture occurs, releasing its contents into the extracellular environment [28,115].

Unlike apoptosis, necrosis is associated with increased inflammation and may contribute to the progression of *M. abscessus* infection. Activation of necrosis in infected macrophages leads to the release of DAMPs, such as HMGB1 and ATP, which amplify the inflammatory response and may favor the dissemination of the bacteria to other tissues. This pathway can also be exploited by the pathogen to escape the intracellular compartment and avoid degradation in phagolysosomes, promoting bacterial dissemination in the organism [110,116].

This complex interaction between *M. abscessus* virulence factors and the host’s immune response is one of the mechanisms that explains the difficulty in controlling this infection and its association with significant tissue damage.

### 3.3. Modulation of Autophagy by M. abscessus and Its Role in Host–Pathogen Interaction

As stated, autophagy is a fundamental cellular process in which damaged organelles and intracellular pathogens are isolated and degraded. Cellular stress and the activation of pathways such as the mTOR inhibitor and AMPK activation increase the host’s ability to isolate and degrade the pathogen, which is encapsulated in autophagosomes. These autophagosomes subsequently fuse with lysosomes to degrade their contents. During infections, the induction of autophagy can help eliminate microorganisms, including *M. abscessus* [117]. However, this premise may not be entirely correct, as *M. abscessus*, like other mycobacteria such as *M. tuberculosis*, has evasion strategies and can modulate the autophagic pathway [117].

Data demonstrate the ability of *M. abscessus* to interfere with the fusion of autophagosomes with lysosomes, a critical step for pathogen degradation. This interference may allow the bacteria to survive within immature autophagic compartments [1]. Furthermore, *M. abscessus* can modulate signaling pathways (such as the mTOR/AMPK pathway) to create an environment that favors its persistence. By altering these signals, the pathogen can reduce the effectiveness of autophagy as an elimination mechanism [118].

The smooth and rough morphotypes of *M. abscessus* appear to interact differently with autophagy. For example, the smooth morphotype tends to inhibit or delay the autophagic response, favoring its intracellular survival, while the rough morphotype can induce more intense inflammatory responses, triggering autophagy [113,119,120]. Interestingly, the rough morphotype is also capable of forming large social phagosomes, which contain numerous bacteria inside. These social phagosomes can mature quickly and fuse with lysosomes; however, despite the acidic and radical environment present in these phagolysosomes, this rough variant can continue to divide rapidly and dominate the defenses of macrophages, resulting in intense autophagy and the induction of cell death, dictating a pro-apoptotic profile [113,119,120].

Therefore, if the autophagic response is too intense or prolonged, the cell may also begin to degrade essential components, such as mitochondria and ribosomes, compromising vital cell functions. In this situation, although the initial activation is an attempt to eliminate the pathogen, excess autophagy can compromise the viability of the host cell itself. Studies indicate that *M. abscessus* can modulate the autophagic process. For example, the rough morphotype tends to induce a more intense inflammatory response, which can favor an exacerbated autophagic activation, while the smooth morphotype can inhibit or delay the maturation of the autophagosome, allowing the persistence of the pathogen [113,119]. When autophagic activity exceeds a critical point, the cell is unable to maintain its homeostasis and is thus driven to death. This cell death by autophagy may be a strategy to eliminate infected cells; however, if the resulting cell bodies are not quickly removed by the immune system, *M. abscessus* may be released into the extracellular environment, facilitating its dissemination and potentially exacerbating the inflammatory response [113].

Although autophagy is generally considered a cell survival mechanism, its excessive activation can lead to autophagic cell death. In *M. abscessus* infections, over-activation of autophagy can result in the degradation of essential organelles, causing collapse of cellular homeostasis and death of the host cell. This cell death may have a bidirectional impact on infection: on the one hand, it may limit bacterial replication by eliminating infected cells; on the other hand, it may release *M. abscessus* into the extracellular environment, facilitating its dissemination and hindering eradication by the immune system. Further studies are needed to clarify whether therapeutic induction of autophagy may be an effective strategy against non-tuberculous mycobacterial infections [113,119].

### 3.4. Modulation of Pyroptosis and Inflammasome Activation by M. abscessus in Infection

During infection, immune cells such as macrophages recognize *M. abscessus* components through receptors like TLR2 and certain NOD-like receptors. This detection can lead to the formation of the NLRP3 inflammasome, a complex that activates caspase-1 [113]. Activation of caspase-1 within the inflammasome results in the cleavage of gasdermin D, whose N-terminal fragment forms pores in the cell membrane. This action culminates in cell lysis and the release of IL-1β, IL-18, and other DAMPs, reinforcing the inflammatory response [121].

**Figure 2:**
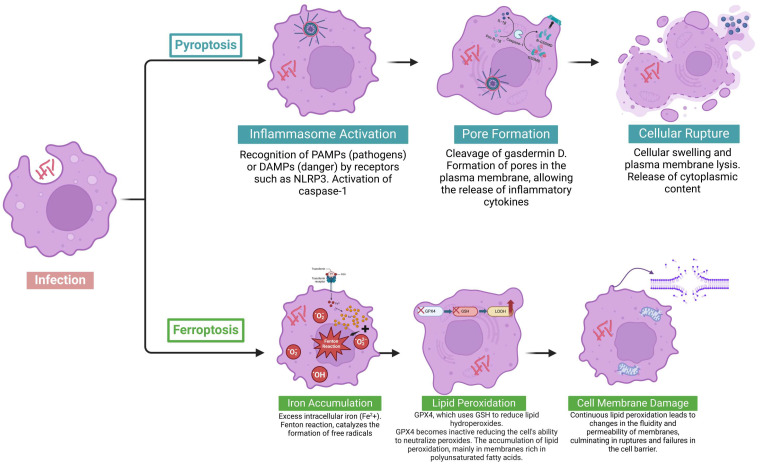
Cell death pathways activated by *M. abscessus* infection: pyroptosis and ferroptosis. After infection by *M. abscessus*, how previously described, how two distinct pathways of cell death, pyroptosis and ferroptosis, are activated during the infection process, highlighting their main stages. In pyroptosis, the process begins with the activation of the inflammasome (inflammasome activation), in which receptors such as NLRP3 detect pathogen-associated molecular patterns (PAMPs) or damage-associated molecular patterns (DAMPs) and promote the activation of caspase-1. Next, gasdermin D is cleaved (pore formation), forming pores in the plasma membrane, which leads to cell rupture and the release of cytoplasmic contents, including pro-inflammatory cytokines, thereby amplifying the inflammatory response. In ferroptosis, intracellular iron accumulates (iron accumulation), promoting Fenton-type reactions that generate free radicals. These radicals induce lipid peroxidation (lipid peroxidation), particularly damaging polyunsaturated fatty acids in the membranes. This results in progressive impairment of membrane integrity (cell membrane damage), leading to functional failure of the cell and death by ferroptosis. Created in BioRender. Pinheiro, R.O. (2025) https://BioRender.com/0zr3igz.

Pyroptosis of infected cells may help eliminate the pathogen’s intracellular niche, limiting *M. abscessus* replication (Figure 2). The release of inflammatory cytokines also attracts other immune cells to the site of infection, intensifying the defense response. However, if the inflammatory response is excessive, tissue damage and the release of viable bacteria may occur, facilitating the spread of the pathogen. Studies suggest that *M. abscessus* can modulate inflammasome activation and the production of IL-1β, contributing to MAB-induced lung pathology by elevating IL-17 production [121,122,123].

During *M. abscessus* infections, activation of pyroptosis may represent an attempt by the immune system to eliminate infected cells and alert the immune system through the release of IL-1β and IL-18. However, pyroptosis may also favor bacterial dissemination, as the resulting cell lysis releases *M. abscessus* into the extracellular milieu, where it can infect new cells. Furthermore, uncontrolled activation of pyroptosis may contribute to lung tissue damage and chronic inflammation associated with persistent non-tuberculous mycobacterial infections [121,123].

### 3.5. Modulation of Ferroptosis and Inflammasome Activation by M. abscessus in Infection

Ferroptosis is a regulated form of cell death characterized by iron accumulation, lipid peroxidation, and failure of antioxidant systems, particularly GPX4 [104]. Although the exact mechanisms behind the induction of ferroptosis by *M. abscessus* are still under investigation, studies suggest that activation of this form of cell death can result in the killing of the bacillus.

The inflammatory response can increase iron uptake and promote the release of intracellular iron from deposits, elevating free iron levels within the cell. Furthermore, the TfR1 is a key mechanism for intracellular iron accumulation. This excess free iron, under conditions of inflammation, catalyzes Fenton-type reactions, generating reactive oxygen species (ROS). This increase in oxidative stress is one of the main triggers for lipid peroxidation [93,95,124,125,126].

*M. abscessus* infection can alter iron homeostasis and trigger an intense inflammatory response that favors iron accumulation and ROS generation. In this way, the pathogen indirectly contributes to creating an environment conducive to ferroptosis [126,127,128] (Figure 2).

If ferroptosis occurs in an exacerbated form in infected cells, it can lead to the death of macrophages or epithelial cells, releasing the pathogen into the extracellular environment. This release may promote the spread of *M. abscessus* and perpetuate local inflammation [129].

Studies show that the induction of ferroptosis exhibits classic morphological aspects found in *M. abscessus* infection, such as mitochondrial alterations, including a reduction in mitochondrial volume, increased cristae density, rupture of the mitochondrial membrane, and the absence of typical signs of apoptosis, such as nuclear condensation or DNA fragmentation [126,129].

These events that can lead to ferroptosis during *M. abscessus* infection—highlighting the modulation of iron metabolism, the increase in ROS, and the failure of antioxidant systems—converge to induce cell death by ferroptosis.

## 4. Conclusions

Understanding the mechanisms of cell death in NTM infections, particularly in the context of *M. abscessus*, reveals a complex interplay between host defense strategies and pathogen evasion mechanisms. Studies demonstrate that these bacteria, naturally found in the environment, are not mere contaminants but opportunistic pathogens capable of colonizing and infecting individuals with chronic lung diseases and other predisposing factors [2,3,4,5,6,130]. During infection, *M. abscessus* manipulates several cell death pathways—including apoptosis, necrosis, pyroptosis, autophagy, and ferroptosis—to favor its survival and dissemination.

Apoptosis, a programmed cell death mechanism that typically eliminates infected cells without triggering inflammation, can be modulated by the pathogen to inhibit efficient macrophage clearance, allowing the bacteria to persist in a protected intracellular niche [35,36,37,38,39,40,41,42,43]. In contrast, necrosis and necroptosis, processes that lead to cell membrane rupture and the release of inflammatory contents, contribute to the intensification of the immune response but also cause tissue damage that may facilitate bacterial dissemination [61,62,63,64,65,66,67].

Autophagy, on the other hand, is a dual mechanism. Under controlled conditions, it recycles damaged organelles and eliminates pathogens, thereby contributing to cellular homeostasis. However, *M. abscessus* infection can trigger excessive activation of this pathway. When autophagy exceeds the adaptive threshold, self-digestion of essential components—such as mitochondria, ribosomes, and vital proteins—occurs, resulting in the collapse of homeostasis. This indiscriminate degradation leads to the loss of energy production capacity (due to mitochondrial degradation) and the failure of protein synthesis, thereby compromising critical cellular functions [72,73,74,75,76,77,78,83,84,85,131]. Thus, cell death by autophagy (or autophagic death) emerges because of an exaggerated attempt by the host to eliminate the pathogen, which becomes harmful.

Additionally, ferroptosis—characterized by iron accumulation, lipid peroxidation, and failure of antioxidant systems such as GPX4—can be indirectly promoted during *M. abscessus* infections. Intense inflammation increases iron uptake and the generation of reactive oxygen species (ROS), catalyzing reactions that irreversibly damage cell membranes. This scenario contributes to death by ferroptosis, another mechanism that can favor the release of bacteria and the spread of infection [93,94,95,124,126,127,128].

Therefore, the interaction between *M. abscessus* and cell death pathways illustrates the dual nature of defense mechanisms: while the activation of these pathways can limit pathogen replication and dissemination, their lack of regulation—especially in relation to autophagy—results in the loss of cellular integrity and, paradoxically, facilitates bacterial spread.

One of the main limitations in understanding the mechanisms of cell death induced by *M. abscessus* lies in the lack of detailed information on the specificity of findings for different subspecies. *M. abscessus* is a complex formed by three main subspecies (*M. abscessus* subsp. *abscessus*, *M. abscessus* subsp. *massiliense,* and *M. abscessus* subsp. *bolletii*), which present significant genetic and phenotypic differences, influencing their virulence, antibiotic resistance, and interaction with the host immune system. However, many studies do not clearly specify which subspecies was analyzed, which makes it difficult to generalize the mechanisms of cell death described. This lack of distinction may lead to imprecise interpretations about the role of apoptosis, necrosis, autophagy, and ferroptosis in the pathogenesis of *M. abscessus*, in addition to limiting the development of more targeted therapeutic approaches for each subspecies. Therefore, there is an urgent need for studies that clarify this issue, allowing a more precise understanding of the mechanisms involved in the host response to *M. abscessus* infections.

In summary, understanding the mechanisms of cell death during *M. abscessus* infections highlights an intricate network of immune responses that, although aimed at eliminating the pathogen, can contribute to disease pathogenesis when dysregulated.

Autophagy, which normally promotes cellular recycling and maintenance, can become a self-destructive process when excessively activated, leading to self-digestion and homeostasis collapse. This phenomenon, along with the modulation of other pathways—such as apoptosis, necrosis, pyroptosis, and ferroptosis—emphasizes the importance of maintaining a balance between defense mechanisms and the need for a controlled immune response.

Understanding these processes not only sheds light on the biological and physiological aspects of NTM infections but also paves the way for the development of new therapeutic approaches. Strategies that specifically modulate cell death pathways, restoring homeostatic balance without compromising pathogen elimination, could represent significant advances in the treatment of infections caused by *M. abscessus* and other opportunistic mycobacteria.

## 5. Perspectives

The regulation of cell death mechanisms has emerged as a promising approach to combat mycobacterial infections, and this may be the case for NTMs, such as *M. abscessus* [132]. Given the complexity of the interaction between the bacteria and the host immune system, therapeutic interventions that promote or inhibit specific cell death pathways may be essential to optimize the immune response and contain the spread of the infection [133].

Induction of apoptosis in infected cells may be an effective strategy to limit the persistence of NTMs in the body. Since these bacteria often use evasion mechanisms to avoid elimination by the immune system, selective activation of apoptosis in macrophages may facilitate the release of the bacteria to neighboring uninfected cells, allowing their elimination by secondary phagocytosis [130,134,135]. Compounds that activate the intrinsic pathway of apoptosis, such as agonists of Bcl-2 family proteins (e.g., BH3 mimetics), could be explored in this context [136].

Another approach involves stimulating autophagy, a process that may contribute to the intracellular degradation of mycobacteria [137]. Autophagy inducers, such as rapamycin (mTOR inhibitor), may aid in bacterial elimination within infected macrophages [121]. In addition, activation of autophagy may promote a cellular environment unfavorable to the survival of NTMs, reducing their ability to persist intracellularly [122].

Although some forms of cell death contribute to the elimination of the infection, others may favor bacterial dissemination and worsening of the inflammatory response [123,124,125]. Pyroptosis, for example, is an inflammatory form of cell death that results in the uncontrolled release of intracellular contents, favoring the spread of NTMs to other cells and exacerbating tissue inflammation [126]. Thus, strategies that inhibit caspase-1 activation and gasdermin D-mediated pore formation may reduce the intensity of the inflammatory response without compromising the immune system’s ability to eliminate the infection [92]. Inflammasome inhibitors, such as MCC950 (an NLRP3 inflammasome antagonist), could be evaluated in this context [127].

Furthermore, ferroptosis, characterized by the accumulation of lipid peroxidation and oxidative stress, can be detrimental in lung infections, leading to excessive tissue destruction and favoring bacterial replication [94,128]. Antioxidants and iron metabolism modulators, such as ferrostatin-1 and liproxstatin-1, could be investigated as potential protective agents against ferroptosis-induced damage [129,138,139,140].

Given that different cell death pathways may be actively involved in the immune response against NTMs, combining modulators of these pathways with conventional antibiotics may represent an innovative strategy to improve treatment efficacy [141]. By promoting apoptosis and autophagy, while inhibiting destructive inflammatory mechanisms such as pyroptosis and ferroptosis, it is possible to enhance bacterial elimination without causing excessive damage to host tissues [142,143,144,145,146,147].

In the area of innovation, there is an urgent need for more representative experimental models of NTM infection, including in vitro and in vivo systems that allow us to evaluate how different cell death pathways influence the pathogenesis and persistence of these bacteria in the organism. Three-dimensional models based on organoids or cell co-cultures can help to more faithfully reproduce the lung environment, allowing the identification of more precise therapeutic targets. In addition, the use of gene editing tools, such as CRISPR-Cas9, can help in understanding the regulation of cell death pathways during infection and may help identify new therapeutic targets that optimize the host immune response against these difficult-to-treat infections [148,149,150,151].

In summary, the understanding of the interactions between the different cell death pathways in NTM infections is still incipient, representing a challenge but also an opportunity for significant advances in the research and treatment of these infections. Future studies should focus on the identification of biomarkers that allow distinguishing the different types of cell death during infection and on the search for therapeutic interventions that optimize the host immune response against these difficult-to-treat bacteria.

## Data Availability

No new data were created or analyzed in this study. Data sharing is not applicable to this article.

## References

[1] Falkinham J.O. (2015). Environmental Sources of Nontuberculous Mycobacteria. Clin. Chest. Med..

[2] Feng J.Y., Chen W.C., Chen Y.Y., Su W.J. (2020). Clinical Relevance and Diagnosis of Nontuberculous Mycobacterial Pulmonary Disease in Populations at Risk. J. Formos. Med. Assoc..

[3] Cowman S., Van Ingen J., Griffith D.E., Loebinger M.R. (2019). Non-Tuberculous Mycobacterial Pulmonary Disease. Eur. Respir. J..

[4] Lake M.A., Ambrose L.R., Lipman M.C.I., Lowe D.M. (2016). “Why Me, Why Now?” Using Clinical Immunology and Epidemiology to Explain Who Gets Nontuberculous Mycobacterial Infection. BMC Med..

[5] de Mello K.G.C., Queiroz Mello F.C., Borga L., Rolla V., Duarte R.S., Sampaio E.P., Holland S.M., Prevots D.R., Dalcolmo M.P. (2013). Clinical and Therapeutic Features of Pulmonary Nontuberculous Mycobacterial Disease, Brazil, 1993–2011. Emerg. Infect. Dis..

[6] Gochi M., Takayanagi N., Kanauchi T., Ishiguro T., Yanagisawa T., Sugita Y. (2015). Retrospective Study of the Predictors of Mortality and Radiographic Deterioration in 782 Patients with Nodular/Bronchiectatic *Mycobacterium avium* Complex Lung Disease. BMJ Open.

[7] Qu H.Q., Fisher-Hoch S.P., McCormick J.B. (2011). Molecular Immunity to Mycobacteria: Knowledge from the Mutation and Phenotype Spectrum Analysis of Mendelian Susceptibility to Mycobacterial Diseases. Int. J. Infect. Dis..

[8] Haverkamp M.H., van Dissel J.T., Holland S.M. (2006). Human Host Genetic Factors in Nontuberculous Mycobacterial Infection: Lessons from Single Gene Disorders Affecting Innate and Adaptive Immunity and Lessons from Molecular Defects in Interferon-Gamma-Dependent Signaling. Microbes Infect..

[9] Rosenzweig S.D., Holland S.M. (2005). Defects in the Interferon-γ and Interleukin-12 Pathways. Immunol. Rev..

[10] Holland S.M. (2000). Treatment of Infections in the Patient with Mendelian Susceptibility to Mycobacterial Infection. Microbes Infect..

[11] Guide S.V., Holland S.M. (2002). Host Susceptibility Factors in Mycobacterial Infection. Genetics and Body Morphotype. Infect. Dis. Clin. N. Am..

[12] Furuya E.Y., Paez A., Srinivasan A., Cooksey R., Augenbraun M., Baron M., Brudney K., Della-Latta P., Estivariz C., Fischer S. (2008). Outbreak of *Mycobacterium abscessus* Wound Infections among “Lipotourists” from the United States Who Underwent Abdominoplasty in the Dominican Republic. Clin. Infect. Dis..

[13] Eustace K., Jolliffe V., Sahota A., Gholam K. (2016). Cutaneous *Mycobacterium abscessus* Infection Following Hair Transplant. Clin. Exp. Dermatol..

[14] Tuan H.T., Ngoc N.A., Ai L.D., Van Luat N. (2024). Complicated Surgical Site Infection with *Mycobacterium abscessus* After Liposuction and Affections of Corticosteroids in the Treatment Regimen: Three Cases Report and a Systematic Review. Aesthet. Plast. Surg..

[15] Ou Y., Liu D., Feng J., Xu X., Lin T., Zhang Y., Luo L., Wu M., Cui Y. (2024). Subcutaneous Infection Caused by *Mycobacterium abscessus* Following Botulinum Toxin Injections: A Case Report and Literature Review. J. Cosmet. Dermatol..

[16] Pace V., Antinolfi P., Borroni E., Cirillo D.M., Cenci E., Piersimoni C., Cardaccia A., Nofri M., Papalini C., Petruccelli R. (2019). Treating Primary Arthroprosthesis Infection Caused by *Mycobacterium abscessus* Subsp. abscessus. Case Rep. Infect. Dis..

[17] Appelgren P., Farnebo F., Dotevall L., Studahl M., Jönsson B., Petrini B. (2008). Late-Onset Posttraumatic Skin and Soft-Tissue Infections Caused by Rapid-Growing Mycobacteria in Tsunami Survivors. Clin. Infect. Dis..

[18] Dahl V.N., Mølhave M., Fløe A., van Ingen J., Schön T., Lillebaek T., Andersen A.B., Wejse C. (2022). Global Trends of Pulmonary Infections with Nontuberculous Mycobacteria: A Systematic Review. Int. J. Infect. Dis..

[19] Prieto M.D., Alam M.E., Franciosi A.N., Quon B.S. (2023). Global Burden of Nontuberculous Mycobacteria in the Cystic Fibrosis Population: A Systematic Review and Meta-Analysis. ERJ Open Res..

[20] Moore M., Frerichs J.B. (1953). An Unusual Acid-Fast Infection of the Knee with Subcutaneous, Abscess-like Lesions of the Gluteal Region; Report of a Case with a Study of the Organism, *Mycobacterium abscessus*, n. Sp. J. Investig. Dermatol..

[21] Bryant J.M., Grogono D.M., Greaves D., Foweraker J., Roddick I., Inns T., Reacher M., Haworth C.S., Curran M.D., Harris S.R. (2013). Whole-Genome Sequencing to Identify Transmission of *Mycobacterium abscessus* between Patients with Cystic Fibrosis: A Retrospective Cohort Study. Lancet.

[22] Tortoli E., Kohl T.A., Brown-Elliott B.A., Trovato A., Leão S.C., Garcia M.J., Vasireddy S., Turenne C.Y., Griffith D.E., Philley J.V. (2016). Emended Description of *Mycobacterium abscessus*, *Mycobacterium abscessus* subsp. *abscessus* and *Mycobacterium abscessus* subsp. *bolletii* and Designation of *Mycobacterium abscessus* subsp. *massiliense* Comb. Nov. Int. J. Syst. Evol. Microbiol..

[23] Leao S.C., Tortoli E., Paul Euzé J., Garcia M.J. (2011). Proposal That *Mycobacterium massiliense* and *Mycobacterium bolletii* Be United and Reclassified as *Mycobacterium abscessus* subsp. *bolletii* Comb. Nov., Designation of *Mycobacterium abscessus* subsp. *abscessus* Subsp. Nov. and Emended Description of *Mycobacterium abscessus*. Int. J. Syst. Evol. Microbiol..

[24] Jones R.S., Shier K.L., Master R.N., Bao J.R., Clark R.B. (2019). Current Significance of the *Mycobacterium chelonae-abscessus* Group. Diagn. Microbiol. Infect. Dis..

[25] Simmon K.E., Brown-Elliott B.A., Ridge P.G., Durtschi J.D., Mann L.B., Slechta E.S., Steigerwalt A.G., Moser B.D., Whitney A.M., Brown J.M. (2011). *Mycobacterium chelonae-abscessus* Complex Associated with Sinopulmonary Disease, Northeastern USA. Emerg. Infect. Dis..

[26] Abdelaal H.F.M., Chan E.D., Young L., Baldwin S.L., Coler R.N. (2022). *Mycobacterium abscessus*: It’s Complex. Microorganisms.

[27] Clary G., Sasindran S.J., Nesbitt N., Mason L., Cole S., Azad A., McCoy K., Schlesinger L.S., Hall-Stoodley L. (2018). *Mycobacterium abscessus* Smooth and Rough Morphotypes Form Antimicrobial-Tolerant Biofilm Phenotypes but Are Killed by Acetic Acid. Antimicrob. Agents Chemother..

[28] Bernut A., Herrmann J.-L., Kissa K., Dubremetz J.-F., Gaillard J.-L., Lutfalla G., Kremer L. (2014). *Mycobacterium abscessus* Cording Prevents Phagocytosis and Promotes Abscess Formation. Proc. Natl. Acad. Sci. USA.

[29] Helguera-Repetto A.C., Chacon-Salinas R., Cerna-Cortes J.F., Rivera-Gutierrez S., Ortiz-Navarrete V., Estrada-Garcia I., Gonzalez-Y-Merchand J.A. (2014). Differential Macrophage Response to Slow- and Fast-Growing Pathogenic Mycobacteria. BioMed Res. Int..

[30] Newton K., Strasser A., Kayagaki N., Dixit V.M. (2024). Cell Death. Cell.

[31] Bertheloot D., Latz E., Franklin B.S. (2021). Necroptosis, Pyroptosis and Apoptosis: An Intricate Game of Cell Death. Cell. Mol. Immunol..

[32] Wanford J.J., Hachani A., Odendall C. (2022). Reprogramming of Cell Death Pathways by Bacterial Effectors as a Widespread Virulence Strategy. Infect. Immun..

[33] Place D.E., Lee S., Kanneganti T.-D. (2021). PANoptosis in Microbial Infection. Curr. Opin. Microbiol..

[34] Bittencourt T.L., da Silva Prata R.B., de Andrade Silva B.J., de Mattos Barbosa M.G., Dalcolmo M.P., Pinheiro R.O. (2021). Autophagy as a Target for Drug Development of Skin Infection Caused by Mycobacteria. Front. Immunol..

[35] Elmore S. (2007). Apoptosis: A Review of Programmed Cell Death. Toxicol. Pathol..

[36] D’Arcy M.S. (2019). Cell Death: A Review of the Major Forms of Apoptosis, Necrosis and Autophagy. Cell. Biol. Int..

[37] Igney F.H., Krammer P.H. (2002). Death and Anti-Death: Tumour Resistance to Apoptosis. Nat. Rev. Cancer.

[38] Yuan J., Ofengeim D. (2024). A Guide to Cell Death Pathways. Nat. Rev. Mol. Cell. Biol..

[39] Green D.R. (2022). The Mitochondrial Pathway of Apoptosis Part II: The BCL-2 Protein Family. Cold Spring Harb. Perspect. Biol..

[40] Lakhani S.A., Masud A., Kuida K., Porter G.A., Booth C.J., Mehal W.Z., Inayat I., Flavell R.A. (2006). Caspases 3 and 7: Key Mediators of Mitochondrial Events of Apoptosis. Science (1979).

[41] Glover H.L., Schreiner A., Dewson G., Tait S.W.G. (2024). Mitochondria and Cell Death. Nat. Cell. Biol..

[42] Rudd-Schmidt J.A., Trapani J.A., Voskoboinik I. (2019). Distinguishing Perforin-Mediated Lysis and Granzyme-Dependent Apoptosis. Methods Enzymol..

[43] Kiselevsky D.B. (2020). Granzymes and Mitochondria. Biochemistry.

[44] Martinvalet D., Zhu P., Lieberman J. (2005). Granzyme A Induces Caspase-Independent Mitochondrial Damage, a Required First Step for Apoptosis. Immunity.

[45] Ben-Abdallah M., Sturny-Leclère A., Avé P., Louise A., Moyrand F., Weih F., Janbon G., Mémet S. (2012). Fungal-Induced Cell Cycle Impairment, Chromosome Instability and Apoptosis via Differential Activation of NF-ΚB. PLoS. Pathog..

[46] Jie W., Rui-Fen Z., Zhong-Xiang H., Yan W., Wei-Na L., Yong-Ping M., Jing S., Jing-Yi C., Wan-Hong L., Xiao-Hua H. (2022). Inhibition of Cell Proliferation by Tas of Foamy Viruses through Cell Cycle Arrest or Apoptosis Underlines the Different Mechanisms of Virus-Host Interactions. Virulence.

[47] Zhou X., Jiang W., Liu Z., Liu S., Liang X. (2017). Virus Infection and Death Receptor-Mediated Apoptosis. Viruses.

[48] Selvaraj C., Vierra M., Dinesh D.C., Abhirami R., Singh S.K. (2021). Structural Insights of Macromolecules Involved in Bacteria-Induced Apoptosis in the Pathogenesis of Human Diseases. Adv. Protein Chem. Struct. Biol..

[49] Krammer P.H., Behrmann I., Daniel P., Dhein J., Debatin K.-M. (1994). Regulation of Apoptosis in the Immune System. Curr. Opin. Immunol..

[50] Ji W., Xin Y., Zhang L., Liu X. (2020). ALG2 Influences T Cell Apoptosis by Regulating FASLG Intracellular Transportation. Biochem. J..

[51] Pol J.G., Caudana P., Paillet J., Piaggio E., Kroemer G. (2020). Effects of Interleukin-2 in Immunostimulation and Immunosuppression. J. Exp. Med..

[52] Katzman S.D., Hoyer K.K., Dooms H., Gratz I.K., Rosenblum M.D., Paw J.S., Isakson S.H., Abbas A.K. (2011). Opposing Functions of IL-2 and IL-7 in the Regulation of Immune Responses. Cytokine.

[53] Morana O., Wood W., Gregory C.D. (2022). The Apoptosis Paradox in Cancer. Int. J. Mol. Sci..

[54] Zhou Z., He H., Wang K., Shi X., Wang Y., Su Y., Wang Y., Li D., Liu W., Zhang Y. (2020). Granzyme A from Cytotoxic Lymphocytes Cleaves GSDMB to Trigger Pyroptosis in Target Cells. Science (1979).

[55] Bellone M. (2000). Apoptosis, Cross-Presentation, and the Fate of the Antigen Specific Immune Response. Apoptosis.

[56] Van Zanten J., Hospers G.A.P., Harmsen M.C., The T.H., Mulder N.H., De Leij L.F.M.H. (2002). Dendritic Cells Present an Intracellular Viral Antigen Derived from Apoptotic Cells and Induce a T-Cell Response. Scand. J. Immunol..

[57] Mahoney J.A., Rosen A. (2005). Apoptosis and Autoimmunity. Curr. Opin. Immunol..

[58] Mountz J.D., Wu J., Cheng J., Zhou T. (1994). Autoimmune Disease. a Problem of Defective Apoptosis. Arthritis Rheum..

[59] Ravirajan C.T., Pittoni V., Isenberg D.A. (1999). Apoptosis in Human Autoimmune Diseases. Int. Rev. Immunol..

[60] Wong R.S. (2011). Apoptosis in Cancer: From Pathogenesis to Treatment. J. Exp. Clin. Cancer Res..

[61] Conrad M., Angeli J.P.F., Vandenabeele P., Stockwell B.R. (2016). Regulated Necrosis: Disease Relevance and Therapeutic Opportunities. Nat. Rev. Drug Discov..

[62] Festjens N., Vanden Berghe T., Vandenabeele P. (2006). Necrosis, a Well-Orchestrated Form of Cell Demise: Signalling Cascades, Important Mediators and Concomitant Immune Response. Biochim. Biophys. Acta. (BBA)-Bioenerg..

[63] Linkermann A., Green D.R. (2014). Necroptosis. N. Engl. J. Med..

[64] Berghe T.V., Linkermann A., Jouan-Lanhouet S., Walczak H., Vandenabeele P. (2014). Regulated Necrosis: The Expanding Network of Non-Apoptotic Cell Death Pathways. Nat. Rev. Mol. Cell Biol..

[65] Tonnus W., Meyer C., Paliege A., Belavgeni A., von Mässenhausen A., Bornstein S.R., Hugo C., Becker J.U., Linkermann A. (2019). The Pathological Features of Regulated Necrosis. J. Pathol..

[66] Pasparakis M., Vandenabeele P. (2015). Necroptosis and Its Role in Inflammation. Nature.

[67] Grootjans S., Vanden Berghe T., Vandenabeele P. (2017). Initiation and Execution Mechanisms of Necroptosis: An Overview. Cell Death Differ..

[68] Vakkila J., Lotze M.T. (2004). Inflammation and Necrosis Promote Tumour Growth. Nat. Rev. Immunol..

[69] Mocarski E.S. (2023). Programmed Necrosis in Host Defense. Curr Trop Microbiol Immunol..

[70] Murao A., Aziz M., Wang H., Brenner M., Wang P. (2021). Release Mechanisms of Major DAMPs. Apoptosis.

[71] Galluzzi L., Kepp O., Chan F.K.-M., Kroemer G. (2017). Necroptosis: Mechanisms and Relevance to Disease. Annu. Rev. Pathol. Mech. Dis..

[72] Glick D., Barth S., Macleod K.F. (2010). Autophagy: Cellular and Molecular Mechanisms. J. Pathol..

[73] Ma T., Yang L., Zhang B., Lv X., Gong F., Yang W. (2023). Hydrogen Inhalation Enhances Autophagy via the AMPK/MTOR Pathway, Thereby Attenuating Doxorubicin-Induced Cardiac Injury. Int. Immunopharmacol..

[74] Khalil M.I., Ali M.M., Holail J., Houssein M. (2023). Growth or Death? Control of Cell Destiny by MTOR and Autophagy Pathways. Prog. Biophys. Mol. Biol..

[75] Alers S., Löffler A.S., Wesselborg S., Stork B. (2012). Role of AMPK-MTOR-Ulk1/2 in the Regulation of Autophagy: Cross Talk, Shortcuts, and Feedbacks. Mol. Cell Biol..

[76] Hamaoui D., Subtil A. (2022). ATG16L1 Functions in Cell Homeostasis beyond Autophagy. FEBS J..

[77] Changotra H., Kaur S., Yadav S.S., Gupta G.L., Parkash J., Duseja A. (2022). ATG5: A Central Autophagy Regulator Implicated in Various Human Diseases. Cell Biochem. Funct..

[78] Metlagel Z., Otomo C., Ohashi K., Takaesu G., Otomo T. (2014). Structural Insights into E2–E3 Interaction for LC3 Lipidation. Autophagy.

[79] Bortoluci K.R., Medzhitov R. (2010). Control of Infection by Pyroptosis and Autophagy: Role of TLR and NLR. Cell. Mol. Life Sci..

[80] Huyghe J., Priem D., Bertrand M.J.M. (2023). Cell Death Checkpoints in the TNF Pathway. Trends Immunol..

[81] Wu W., Wang X., Sun Y., Berleth N., Deitersen J., Schlütermann D., Stuhldreier F., Wallot-Hieke N., José Mendiburo M., Cox J. (2021). TNF-Induced Necroptosis Initiates Early Autophagy Events via RIPK3-Dependent AMPK Activation, but Inhibits Late Autophagy. Autophagy.

[82] Shin D.-M., Jeon B.-Y., Lee H.-M., Jin H.S., Yuk J.-M., Song C.-H., Lee S.-H., Lee Z.-W., Cho S.-N., Kim J.-M. (2010). *Mycobacterium tuberculosis* Eis Regulates Autophagy, Inflammation, and Cell Death through Redox-Dependent Signaling. PLoS Pathog..

[83] Liu S., Yao S., Yang H., Liu S., Wang Y. (2023). Autophagy: Regulator of Cell Death. Cell Death Dis..

[84] Noguchi M., Hirata N., Tanaka T., Suizu F., Nakajima H., Chiorini J.A. (2020). Autophagy as a Modulator of Cell Death Machinery. Cell Death Dis..

[85] Mahapatra K.K., Mishra S.R., Behera B.P., Patil S., Gewirtz D.A., Bhutia S.K. (2021). The Lysosome as an Imperative Regulator of Autophagy and Cell Death. Cell. Mol. Life Sci..

[86] Arumugam P., Shankaran D., Bothra A., Gandotra S., Rao V. (2019). The MmpS6-MmpL6 Operon Is an Oxidative Stress Response System Providing Selective Advantage to *Mycobacterium tuberculosis* in Stress. J. Infect. Dis..

[87] Shankaran D., Arumugam P., Vasanthakumar R.P., Singh A., Bothra A., Gandotra S., Rao V. (2022). Modern Clinical *Mycobacterium tuberculosis* Strains Leverage Type I IFN Pathway for a Proinflammatory Response in the Host. J. Immunol..

[88] Broz P., Dixit V.M. (2016). Inflammasomes: Mechanism of Assembly, Regulation and Signalling. Nat. Rev. Immunol..

[89] Li L., Dickinson M.S., Coers J., Miao E.A. (2023). Pyroptosis in Defense against Intracellular Bacteria. Semin. Immunol..

[90] Xiao C., Cao S., Li Y., Luo Y., Liu J., Chen Y., Bai Q., Chen L. (2024). Pyroptosis in Microbial Infectious Diseases. Mol. Biol. Rep..

[91] Shi J., Zhao Y., Wang K., Shi X., Wang Y., Huang H., Zhuang Y., Cai T., Wang F., Shao F. (2015). Cleavage of GSDMD by Inflammatory Caspases Determines Pyroptotic Cell Death. Nature.

[92] Kovacs S.B., Miao E.A. (2017). Gasdermins: Effectors of Pyroptosis. Trends Cell Biol..

[93] Vasudevan S.O., Behl B., Rathinam V.A. (2023). Pyroptosis-Induced Inflammation and Tissue Damage. Semin. Immunol..

[94] Stockwell B.R., Friedmann Angeli J.P., Bayir H., Bush A.I., Conrad M., Dixon S.J., Fulda S., Gascón S., Hatzios S.K., Kagan V.E. (2017). Ferroptosis: A Regulated Cell Death Nexus Linking Metabolism, Redox Biology, and Disease. Cell.

[95] Dixon S.J., Lemberg K.M., Lamprecht M.R., Skouta R., Zaitsev E.M., Gleason C.E., Patel D.N., Bauer A.J., Cantley A.M., Yang W.S. (2012). Ferroptosis: An Iron-Dependent Form of Nonapoptotic Cell Death. Cell.

[96] Ursini F., Maiorino M. (2020). Lipid Peroxidation and Ferroptosis: The Role of GSH and GPx4. Free Radic. Biol. Med..

[97] Henning Y., Blind U.S., Larafa S., Matschke J., Fandrey J. (2022). Hypoxia Aggravates Ferroptosis in RPE Cells by Promoting the Fenton Reaction. Cell Death Dis..

[98] Feng H., Schorpp K., Jin J., Yozwiak C.E., Hoffstrom B.G., Decker A.M., Rajbhandari P., Stokes M.E., Bender H.G., Csuka J.M. (2020). Transferrin Receptor Is a Specific Ferroptosis Marker. Cell Rep..

[99] Park E., Chung S.W. (2019). ROS-Mediated Autophagy Increases Intracellular Iron Levels and Ferroptosis by Ferritin and Transferrin Receptor Regulation. Cell Death Dis..

[100] Wang Y., Zhang M., Bi R., Su Y., Quan F., Lin Y., Yue C., Cui X., Zhao Q., Liu S. (2022). ACSL4 Deficiency Confers Protection against Ferroptosis-Mediated Acute Kidney Injury. Redox Biol..

[101] Cui J., Wang Y., Tian X., Miao Y., Ma L., Zhang C., Xu X., Wang J., Fang W., Zhang X. (2023). LPCAT3 Is Transcriptionally Regulated by YAP/ZEB/EP300 and Collaborates with ACSL4 and YAP to Determine Ferroptosis Sensitivity. Antioxid. Redox Signal..

[102] Liu J., Kang R., Tang D. (2022). Signaling Pathways and Defense Mechanisms of Ferroptosis. FEBS J..

[103] Yang W.S., Kim K.J., Gaschler M.M., Patel M., Shchepinov M.S., Stockwell B.R. (2016). Peroxidation of Polyunsaturated Fatty Acids by Lipoxygenases Drives Ferroptosis. Proc. Natl. Acad. Sci. USA.

[104] Whang J., Back Y.W., Lee K.-I., Fujiwara N., Paik S., Choi C.H., Park J.-K., Kim H.-J. (2017). *Mycobacterium abscessus* Glycopeptidolipids Inhibit Macrophage Apoptosis and Bacterial Spreading by Targeting Mitochondrial Cyclophilin D. Cell Death Dis..

[105] Shin D.M., Yang C.S., Yuk J.M., Lee J.Y., Kim K.H., Shin S.J., Takahara K., Lee S.J., Jo E.K. (2008). *Mycobacterium abscessus* Activates the Macrophage Innate Immune Response via a Physical and Functional Interaction between TLR2 and Dectin-1. Cell. Microbiol..

[106] Blanco-Conde S., González-Cortés C., López-Medrano R., Carazo-Fernández L., Diez-Tascón C., Marcos-Benavides M.F., Rivero-Lezcano O.M. (2022). *Mycobacterium abscessus* Infected Neutrophils as an In Vitro Model for Bronchiectasis. Neutrophils Prevent Mycobacterial Aggregation. Arch. Bronconeumol..

[107] Ahn J.-H., Jung D.-H., Kim D.-Y., Lee T.-S., Kim Y.-J., Lee Y.-J., Seo I.-S., Kim W.-G., Cho Y.J., Shin S.J. (2024). Impact of IL-1β on Lung Pathology Caused by *Mycobacterium abscessus* Infection and Its Association with IL-17 Production. Microbes Infect..

[108] Sheng S., Xin L., Yam J.K.H., Salido M.M., Khong N.Z.J., Liu Q., Chea R.A., Li H.Y., Yang L., Liang Z.-X. (2019). The MapZ-Mediated Methylation of Chemoreceptors Contributes to Pathogenicity of *Pseudomonas aeruginosa*. Front. Microbiol..

[109] Bates N.A., Rodriguez R., Drwich R., Ray A., Stanley S.A., Penn B.H. (2024). Reactive Oxygen Detoxification Contributes to *Mycobacterium abscessus* Antibiotic Survival. bioRxiv.

[110] Kim B.R., Kim B.J., Kook Y.H., Kim B.J. (2019). Phagosome Escape of Rough *Mycobacterium abscessus* Strains in Murine Macrophage via Phagosomal Rupture Can Lead to Type I Interferon Production and Their Cell-to-Cell Spread. Front. Immunol..

[111] Bonay M., Roux A.-L., Floquet J., Retory Y., Herrmann J.-L., Lofaso F., Deramaudt T. (2015). Caspase-Independent Apoptosis in Infected Macrophages Triggered by Sulforaphane via Nrf2/P38 Signaling Pathways. Cell Death Discov..

[112] Parmar S., Tocheva E.I. (2023). The Cell Envelope of *Mycobacterium abscessus* and Its Role in Pathogenesis. PLoS Pathog..

[113] Roux A.L., Viljoen A., Bah A., Simeone R., Bernut A., Laencina L., Deramaudt T., Rottman M., Gaillard J.L., Majlessi L. (2016). The Distinct Fate of Smooth and Rough *Mycobacterium abscessus* Variants inside Macrophages. Open Biol..

[114] Catherinot E., Clarissou J., Etienne G., Ripoll F., Emile J.-F., Daffé M., Perronne C., Soudais C., Gaillard J.-L., Rottman M. (2007). Hypervirulence of a Rough Variant of the *Mycobacterium abscessus* Type Strain. Infect. Immun..

[115] PETRINI B. (2006). *Mycobacterium abscessus*: An Emerging Rapid-growing Potential Pathogen. Apmis.

[116] Leestemaker-Palmer A.L., Bermudez L.E. (2023). *Mycobacterium abscessus* Infection Results in Decrease of Oxidative Metabolism of Lung Airways Cells and Relaxation of the Epithelial Mucosal Tight Junctions. Tuberculosis.

[117] Feng Z., Bai X., Wang T., Garcia C., Bai A., Li L., Honda J.R., Nie X., Chan E.D. (2020). Differential Responses by Human Macrophages to Infection With *Mycobacterium tuberculosis* and Non-Tuberculous Mycobacteria. Front. Microbiol..

[118] Quan H., Chung H., Je S., Hong J.J., Kim B.-J., Na Y.R., Seok S.H. (2023). Pyruvate Dehydrogenase Kinase Inhibitor Dichloroacetate Augments Autophagy Mediated Constraining the Replication of *Mycobacteroides massiliense* in Macrophages. Microbes Infect..

[119] Kim S.W., Subhadra B., Whang J., Back Y.W., Bae H.S., Kim H.J., Choi C.H. (2017). Clinical *Mycobacterium abscessus* Strain Inhibits Autophagy Flux and Promotes Its Growth in Murine Macrophages. Pathog. Dis..

[120] Viljoen A., Herrmann J.L., Onajole O.K., Stec J., Kozikowski A.P., Kremer L. (2017). Controlling Extra- and Intramacrophagic *Mycobacterium abscessus* by Targeting Mycolic Acid Transport. Front. Cell. Infect. Microbiol..

[121] Shamaei M., Mirsaeidi M. (2021). Nontuberculous Mycobacteria, Macrophages, and Host Innate Immune Response. Infect. Immun..

[122] Silwal P., Kim I.S., Jo E.-K. (2021). Autophagy and Host Defense in Nontuberculous Mycobacterial Infection. Front. Immunol..

[123] Jorgensen I., Rayamajhi M., Miao E.A. (2017). Programmed Cell Death as a Defence against Infection. Nat. Rev. Immunol..

[124] Ratnatunga C.N., Tungatt K., Proietti C., Halstrom S., Holt M.R., Lutzky V.P., Price P., Doolan D.L., Bell S.C., Field M.A. (2022). Characterizing and Correcting Immune Dysfunction in Non-Tuberculous Mycobacterial Disease. Front. Immunol..

[125] Hu W., Koch B.E.V., Lamers G.E.M., Forn-Cuní G., Spaink H.P. (2022). Specificity of the Innate Immune Responses to Different Classes of Non-Tuberculous Mycobacteria. Front. Immunol..

[126] Liu X., Zhang Z., Ruan J., Pan Y., Magupalli V.G., Wu H., Lieberman J. (2016). Inflammasome-Activated Gasdermin D Causes Pyroptosis by Forming Membrane Pores. Nature.

[127] Coll R.C., Robertson A.A.B., Chae J.J., Higgins S.C., Muñoz-Planillo R., Inserra M.C., Vetter I., Dungan L.S., Monks B.G., Stutz A. (2015). A Small-Molecule Inhibitor of the NLRP3 Inflammasome for the Treatment of Inflammatory Diseases. Nat. Med..

[128] Bagayoko S., Meunier E. (2022). Emerging Roles of Ferroptosis in Infectious Diseases. FEBS J..

[129] Sun S., Shen J., Jiang J., Wang F., Min J. (2023). Targeting Ferroptosis Opens New Avenues for the Development of Novel Therapeutics. Signal Transduct. Target. Ther..

[130] Bar-Oz M., Meir M., Barkan D. (2022). Virulence-Associated Secretion in *Mycobacterium abscessus*. Front. Immunol..

[131] Klionsky D.J., Petroni G., Amaravadi R.K., Baehrecke E.H., Ballabio A., Boya P., Bravo-San Pedro J.M., Cadwell K., Cecconi F., Choi A.M.K. (2021). Autophagy in Major Human Diseases. EMBO J..

[132] Nisa A., Kipper F.C., Panigrahy D., Tiwari S., Kupz A., Subbian S. (2022). Different Modalities of Host Cell Death and Their Impact on *Mycobacterium tuberculosis* Infection. Am. J. Physiol. Cell Physiol..

[133] Awuh J.A., Flo T.H. (2017). Molecular Basis of Mycobacterial Survival in Macrophages. Cell. Mol. Life Sci..

[134] Ochoa A.E., Congel J.H., Corley J.M., Janssen W.J., Nick J.A., Malcolm K.C., Hisert K.B. (2023). Dectin-1-Independent Macrophage Phagocytosis of *Mycobacterium abscessus*. Int. J. Mol. Sci..

[135] Behar S.M., Martin C.J., Booty M.G., Nishimura T., Zhao X., Gan H.-X., Divangahi M., Remold H.G. (2011). Apoptosis Is an Innate Defense Function of Macrophages against *Mycobacterium tuberculosis*. Mucosal. Immunol..

[136] Kale J., Osterlund E.J., Andrews D.W. (2018). BCL-2 Family Proteins: Changing Partners in the Dance towards Death. Cell Death Differ..

[137] Gutierrez M.G., Master S.S., Singh S.B., Taylor G.A., Colombo M.I., Deretic V. (2004). Autophagy Is a Defense Mechanism Inhibiting BCG and *Mycobacterium tuberculosis* Survival in Infected Macrophages. Cell.

[138] Xiao Z., Kong B., Fang J., Qin T., Dai C., Shuai W., Huang H. (2021). Ferrostatin-1 Alleviates Lipopolysaccharide-Induced Cardiac Dysfunction. Bioengineered.

[139] Tong J., Lan X., Zhang Z., Liu Y., Sun D., Wang X., Ou-Yang S., Zhuang C., Shen F., Wang P. (2023). Ferroptosis Inhibitor Liproxstatin-1 Alleviates Metabolic Dysfunction-Associated Fatty Liver Disease in Mice: Potential Involvement of PANoptosis. Acta Pharmacol. Sin..

[140] Fan B.-Y., Pang Y.-L., Li W.-X., Zhao C.-X., Zhang Y., Wang X., Ning G.-Z., Kong X.-H., Liu C., Yao X. (2021). Liproxstatin-1 Is an Effective Inhibitor of Oligodendrocyte Ferroptosis Induced by Inhibition of Glutathione Peroxidase 4. Neural Regen. Res..

[141] Tanouchi Y., Lee A.J., Meredith H., You L. (2013). Programmed Cell Death in Bacteria and Implications for Antibiotic Therapy. Trends Microbiol..

[142] Wang B., Wang Y., Zhang J., Hu C., Jiang J., Li Y., Peng Z. (2023). ROS-Induced Lipid Peroxidation Modulates Cell Death Outcome: Mechanisms behind Apoptosis, Autophagy, and Ferroptosis. Arch. Toxicol..

[143] Zhou B., Liu J., Kang R., Klionsky D.J., Kroemer G., Tang D. (2020). Ferroptosis Is a Type of Autophagy-Dependent Cell Death. Semin. Cancer Biol..

[144] Pang Q., Wang P., Pan Y., Dong X., Zhou T., Song X., Zhang A. (2022). Irisin Protects against Vascular Calcification by Activating Autophagy and Inhibiting NLRP3-Mediated Vascular Smooth Muscle Cell Pyroptosis in Chronic Kidney Disease. Cell Death Dis..

[145] Tang Q., Liu W., Yang X., Tian Y., Chen J., Hu Y., Fu N. (2022). ATG5-Mediated Autophagy May Inhibit Pyroptosis to Ameliorate Oleic Acid-Induced Hepatocyte Steatosis. DNA Cell. Biol..

[146] Deng Z., Wang Y., Liu J., Zhang H., Zhou L., Zhao H., Han Y., Yan S., Dong Z., Wang Y. (2023). WBP2 Restrains the Lysosomal Degradation of GPX4 to Inhibit Ferroptosis in Cisplatin-Induced Acute Kidney Injury. Redox Biol..

[147] Cai X., Hua S., Deng J., Du Z., Zhang D., Liu Z., Khan N.U., Zhou M., Chen Z. (2022). Astaxanthin Activated the Nrf2/HO-1 Pathway to Enhance Autophagy and Inhibit Ferroptosis, Ameliorating Acetaminophen-Induced Liver Injury. ACS Appl. Mater. Interfaces J..

[148] Bhagwat A.C., Patil A.M., Saroj S.D. (2022). CRISPR/Cas 9-Based Editing in the Production of Bioactive Molecules. Mol. Biotechnol..

[149] de Maat V., Stege P.B., Dedden M., Hamer M., van Pijkeren J.-P., Willems R.J.L., van Schaik W. (2019). CRISPR-Cas9-mediated genome editing in vancomycin-resistant *Enterococcus faecium*. FEMS Microbiol. Lett..

[150] Bhattacharjee G., Gohil N., Khambhati K., Mani I., Maurya R., Karapurkar J.K., Gohil J., Chu D.-T., Vu-Thi H., Alzahrani K.J. (2022). Current Approaches in CRISPR-Cas9 Mediated Gene Editing for Biomedical and Therapeutic Applications. J. Control. Release.

[151] Yang X. (2015). Applications of CRISPR-Cas9 Mediated Genome Engineering. Mil. Med. Res..

